# Multiomic Analysis of Neurons with Divergent Projection Patterns Identifies Novel Regulators of Axon Pathfinding

**DOI:** 10.1002/advs.202200615

**Published:** 2022-08-21

**Authors:** Marta Fernández‐Nogales, Maria Teresa López‐Cascales, Verónica Murcia‐Belmonte, Augusto Escalante, Jordi Fernández‐Albert, Rafael Muñoz‐Viana, Angel Barco, Eloísa Herrera

**Affiliations:** ^1^ Instituto de Neurociencias (Consejo Superior de Investigaciones Científicas ‐Universidad Miguel Hernández de Elche, CSIC‐UMH) San Juan de Alicante Av. Santiago Ramón y Cajal s/n Alicante 03550 Spain

**Keywords:** axon guidance, axon midline decisions, chromatin binding, integrins, Lhx, Nrp2, Pou4f, retinal ganglion cells, RGCs, transcriptional regulation, Zic, *γ*−synuclein

## Abstract

Axon pathfinding is a key step in neural circuits formation. However, the transcriptional mechanisms regulating its progression remain poorly understood. The binary decision of crossing or avoiding the midline taken by some neuronal axons during development represents a robust model to investigate the mechanisms that control the selection of axonal trajectories. Here, to identify novel regulators of axon guidance, this work compares the transcriptome and chromatin occupancy profiles of two neuronal subpopulations, ipsilateral (iRGC) and contralateral retinal ganglion cells (cRGC), with similar functions but divergent axon trajectories. These analyses retrieved a number of genes encoding for proteins not previously implicated in axon pathfinding. In vivo functional experiments confirm the implication of some of these candidates in axonal navigation. Among the candidate genes, *γ*‐synuclein is identified as essential for inducing midline crossing. Footprint and luciferase assays demonstrate that this small‐sized protein is regulated by the transcription factor (TF) Pou4f1 in cRGCs. It is also shown that Lhx2/9 are specifically expressed in iRGCs and control a program that partially overlaps with that regulated by Zic2, previously described as essential for iRGC specification. Overall, the analyses identify dozens of new molecules potentially involved in axon guidance and reveal the regulatory logic behind the selection of axonal trajectories.

## Graphical abstract

The results presented here provide genomic screens for regarding genes and regulatory regions involved in axonal trajectory selection and offer new biological insight that may contribute to future experiments addressing how neural circuits may be rewired after damage or be useful to improve the generation of artificial neural circuits growing in brain organoids.

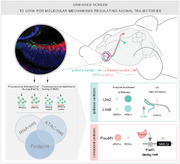

## 1 Introduction

The proper formation of neuronal circuits during embryonic development is critical for brain functioning and the survival of the organism. As a result, aberrant circuit formation may cause a wide variety of birth defects and neurological disorders, such as autism or schizophrenia, that can emerge later in life. The formation of neuronal circuits relies on the coordinated action in time and space of the intracellular signaling proteins, cell‐surface receptors, and secreted cues necessary for initiating and guiding axonal trajectories.^[^ ^1^, ^2^, ^3^ ^]^ The expression of these effector molecules depends on differences in chromatin accessibility and occupancy at both genic and extragenic regions that constrain the action of neuronal type‐specific TFs.^[^ ^4^ ^]^ It is generally accepted that the regulation of gene expression is modular, with particular processes controlled by gene programs in a TF‐dependent manner. The set of active enhancers present in a given type of neuron in a particular moment is highly specific and genome‐wide active enhancers reflect specific cell identities at specific developmental stages.^[^ ^5^, ^6^ ^]^

Many laboratories are currently trying to uncover the transcriptional mechanisms controlling cell fate determination in neural progenitors. Some studies focus on a specific type of TFs, known as terminal selectors, that control mature neural features through the coordinated activation of cell‐type specific enhancers and maintain neuronal identity throughout life.^[^ ^7^, ^8^ ^]^ However, the regulatory mechanisms that specifically control or influence transient and dynamic processes such as axon pathfinding in differentiated but still immature neurons have not been investigated. In fact, although numerous membrane and cytoplasmic proteins that regulate axonal pathfinding have been identified in the last three decades, the epigenetic and transcriptional mechanisms that control their expression remain poorly understood with very few TFs described as directly involved in regulating axon guidance decisions.^[^ ^9^ ^]^

Previous studies have demonstrated the utility of the mammalian visual system as a model to discover general principles ruling the formation of neural circuits.^[^ ^10^ ^]^ In mice, axons from neurons located at the ventro‐temporal (VT) retina avoid the midline at the level of the optic chiasm to project to the ipsilateral side of the brain while axons from the rest of the retina cross the midline projecting in the opposite hemisphere. This binary decision of visual axons, to cross or not the midline, is essential to perceive the world in 3D and represents an excellent paradigm to investigate the mechanisms that enable the connection of neurons with distant targets in the brain during late embryonic development.^[^ ^9^ ^]^

To find novel regulatory mechanisms involved in axon pathfinding, we compared the transcriptome and chromatin occupancy profiles of retinal ganglion cell (RGC) neurons that project to the ipsilateral or the contralateral brain hemisphere. These analyses revealed the regulatory logic behind axon trajectories selection and retrieved numerous genes encoding for proteins not previously implicated in axon guidance. We further analyzed some of these candidates throughout gain and loss‐of‐function (LOF) assays and provide proof‐of‐principle evidence that validates our screens. Our results demonstrate that the newly identified guidance regulators operate in different contexts and open new venues for further research.

## 2 Results

### 2.1 Transcriptomic Signatures of cRGCs and iRGC

**Figure 1:**
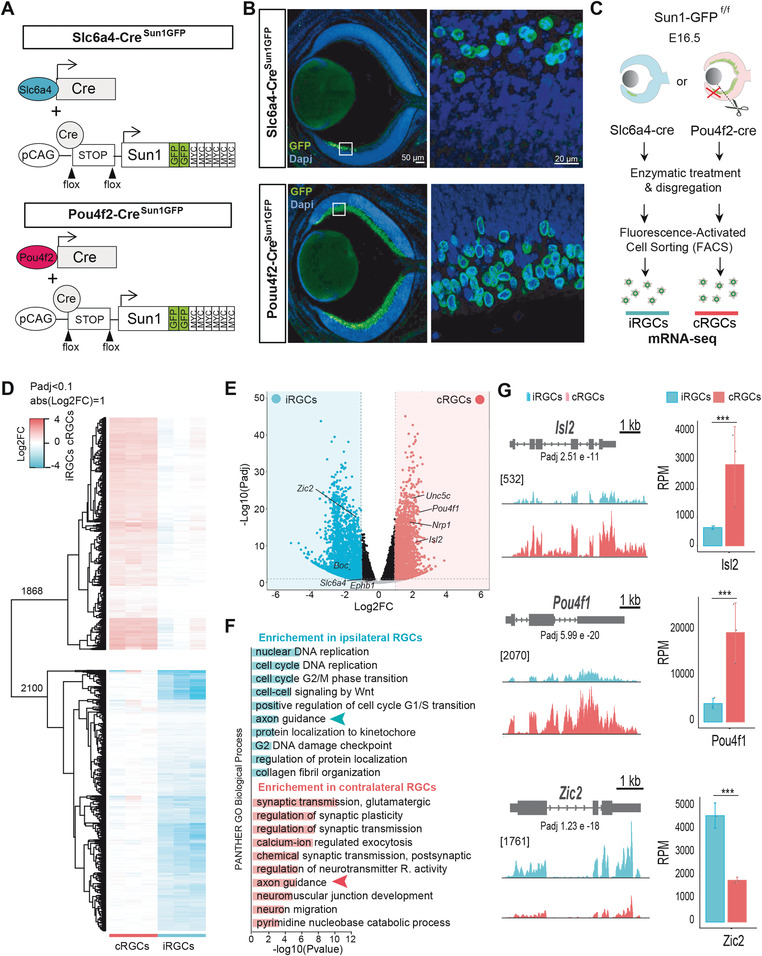
Transcriptome differences among ipsilateral retinal ganglion cells (iRGCs) and contralateral retinal ganglion cells (cRGCs). A) Diagram showing the generation of Pou4f2^Sun1GFP^ and Slc6a4^Sun1GFP^ mouse lines. The cre recombinase‐driver lines Slc6a4‐Cre and Pou4f2‐Cre were crossed with a conditional reporter line that contains a CAG‐[Stop] cassette followed by the nuclear envelope protein Sun1 cDNA sequence fused to GFP. B) Coronal retinal sections from E16.5 Slc6a4‐Cre^Sun1GFP^ or Pou4f2‐Cre^Sun1GFP^ embryos immunostained against GFP and counterstained with DAPI demonstrate that this line specifically labels the nuclear envelope of iRGCs or RGCs, respectively. Scale bar: 50 µm. High magnification of the squared area shows labeled iRGCs. Scale bar: 20 µm. C) Experimental approach used to isolate iRGCs from Slc6a4‐Cre^Sun1GFP^ embryos and cRGCs from Pou4f2‐Cre^Sun1GFP^ embryos after removal of the VT region, to perform mRNA‐seq and compare their transcriptomes. D) Heatmap of fold changes in differentially expressed genes (DEGs) retrieved in the RNA‐seq screen between iRGCs and cRGCs. In the upper part cRGC DEGs refer to the iRGC rowMeans and in the lower part iRGC DEGs refer to the cRGC rowMeans. E) Volcano plot showing the significance and *p*‐value distribution after differential transcript abundance analysis between iRGCs and cRGCs populations. F) Histogram for Panther Gene Ontology (GO) Biological Process terms in DEGs in iRGCs and cRGCs. The bar graphs present the significance of the enrichment. Padj < 0.05 and |log2FC| ≥ 1. G. RNA‐seq profiles of transcription factors known to play key roles in the guidance of cRGCs (Isl2 and Pou4f1) or iRGCs (Zic2) axons. The vertical scale shows counts in reads per million (RPM). Average RPMs for each gene in the iRGC and the cRGC populations are represented in the graphs at the right. (***Padj < 0.001).

To compare the transcriptome of iRGC and cRGCs, we crossed mice bearing the Cre‐dependent reporter cassette CAG‐[Stop]‐Sun1‐GFP inserted in the Rosa26 locus^[^ ^11^, ^23^ ^]^ with two different Cre‐driver lines: Slc6a4‐Cre and Pou4f2‐Cre (**Figure** **1**A). Upon Cre‐mediated recombination, offspring mice express a GFP‐tagged variant of the nuclear envelope protein Sun1 (Sun1‐GFP) in the targeted population.

The serotonin transporter (Slc6a4, aka Sert) is specifically expressed in iRGCs from embryonic day E14 to early postnatal stages.^[^ ^12^ ^]^ Therefore, Slc6a4‐Cre; Sun1‐GFP mice (from now on referred to as Slc6a4^Sun1GFP^) display specific labeling of the nuclear envelope of iRGCs. As expected, GFP signal was restricted to the nuclear envelope of cells located in the peripheral VT region in these mice (Figure 1B). In contrast to iRGCs, there is currently no Cre‐driver strain that allows the specific tagging of the cRGC population. Pou4f2 (aka Brn3b) is a member of the POU‐domain transcription factor family that is expressed in the vast majority of RGCs^[^ ^13^ ^]^ and Pou4f2‐Cre; Sun1‐GFP (from now on referred to as Pou4f2^Sun1GFP^) mouse embryos display green fluorescence in most nuclei at the inner layer of the entire retina (Figure 1B). To specifically isolate cRGCs, avoiding contamination from iRGCs, we dissected retinas from Pou4f2^Sun1GFP^ embryos and removed the VT region (Figure 1C).

Sun1‐GFP^+^ cells were isolated by fluorescence‐activated cell sorting (FACS) from retinas of Slc6a4^SunGFP^ and Pou4f2^SunGFP^ embryos (Figure S1A, Supporting Information) at E16.5, the time when most axons approach the optic chiasm to cross or avoid the midline. Then, RNA was extracted from sorted cells and an RNA‐seq screen was conducted to compare the transcriptome of iRGCs and cRGCs (Figure 1D and Figure S1B,C, Supporting Information). We detected 3968 differentially expressed genes (DEGs) between iRGC and cRGCs. A total of 2100 genes were enriched in the ipsilateral population, while 1868 genes were enriched in the contralateral population (Figure 1D,E and Table S1, Supporting Information).

Mouse RGCs are born following a clockwise wave in the developing retina that initiates at the dorsal‐central part of the optic cup and progresses ventrally and peripherally. As a result, cRGCs are generated between E11 and P0 while iRGCs, which arise from the peripheral VT retina, are born between E14.5 and P0.^[^ ^14^, ^15^ ^]^ Therefore, since we collected the cells at E16.5, the iRGC population is, in average, younger than the cRGC population. Consistent with this, Gene Ontology (GO) enrichment analysis of DEGs retrieved a number of cell cycle‐related terms associated with the iRGC population (Figure 1F). Notably, GO analysis also revealed a significant enrichment in genes associated with the term *Axon Guidance* in the gene sets specific for each RGC subpopulation, which likely relates with the divergent guidance decisions that their axons take at the midline. These DEGs included genes such as *Unc5c, Boc*, and *Nrp1*, encoding axon guidance receptors previously identified as specifically expressed in either one of the subpopulations^[^ ^16^, ^17^, ^18^ ^]^ (Figure 1E). Consistent with previous studies, our screen also retrieved TFs previously identified as specific of cRGCs (e.g., Isl2 and Pou4f1)^[^ ^19^, ^20^ ^]^ or iRGCs (e.g., Zic2)^[^ ^21^ ^]^ (Figure 1G). Overall, these results underscore the efficacy and reliability of our approach to retrieve genes differentially expressed in iRGC and cRGCs when axon guidance decisions are taking place at the midline.

### 2.2 Chromatin Accessibility Differences Between iRGCs and cRGCs

**Figure 2:**
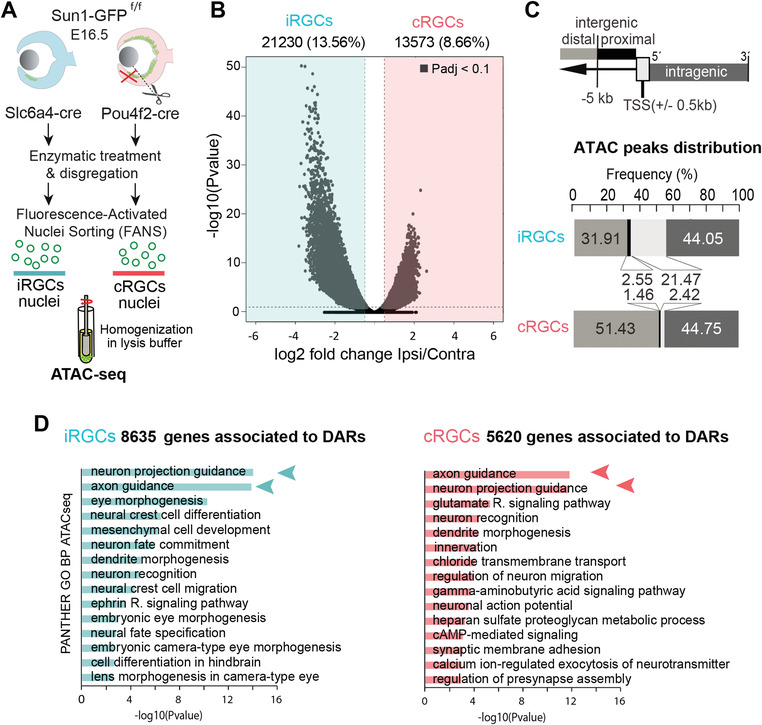
Chromatin accessibility differences between contralateral retinal ganglion cells (cRGCs) and ipsilateral retinal ganglion cells (iRGCs). A) Experimental approach used to isolate iRGCs nuclei from Slc6a4‐Cre^Sun1GFP^ embryos and cRGC nuclei from Pou4f2‐Cre^Sun1GFP^ embryos (with the VT region removed) at E16.5 in order to perform ATAC‐seq assay. B) Volcano plot showing the significance value distribution after differentially accessible region (DAR) analysis between iRGCs and cRGCs populations; upper values indicate number of regions and percentage of all the accessible regions detected (Padj < 0.1). C) ATAC peaks distribution analysis of highly significant DARs at the promoter (TSS +/− 500 pb), proximal enhancer regions (between 0.5 and 5 kb upstream of the TSS), distal enhancer regions (more than 5 kb upstream of the TSS), and in the intragenic regions. The analysis shows the frequency (%) and the number of peaks in each region in the iRGC and cRGC populations. D) Panther Gene Ontology (GO) Biological Process enrichment analysis of DARs in cRGCs and iRGCs. Padj < 0.05 and |log2FC| ≥ 1.

To search for novel regulatory mechanisms involved in axon guidance responses, we next sought differences in chromatin accessibility and occupancy between cRGCs and iRGCs using Assay for Transposase‐Accessible Chromatin coupled to deep sequencing (ATAC‐seq).^[^ ^22^ ^]^ For this, we collected retinas from Slc6a4^SunGFP^ and Pou4f2^SunGFP^ embryos and sorted Sun1‐GFP^+^ nuclei to perform fluorescence‐activated nuclei sorting (FANS) analysis (**Figure** **2**A).^[^ ^23^ ^]^ Again, as described for the RNA‐seq screen, in the case of Pou4f2^SunGFP^ retinas we first removed the peripheral VT part. This screen retrieved more than 150 000 accessible regions in the chromatin of RGCs. Most of these regions were shared by cRGC and iRGC, reflecting the common ontogeny and function of these two neuron populations. In addition, the comparison of cRGCs and iRGCs ATAC‐seq libraries revealed numerous regions displaying differential accessible regions (DARs). The iRGC population presented 21230 specific regions (13,6% of all accessible regions) with enhanced accessibility, whereas cRGCs showed differential accessibility in 13 573 regions (8,7% of total accessible regions) (Figure 2B). Interestingly, the regions displaying enhanced accessibility were more frequently located at the promoter (TSS +/− 500 pb) or at proximal enhancer regions (between 0.5 and 5 kb upstream of the TSS) in iRGC than in cRGCs. In contrast, cRGCs showed more population‐specific DARs in distal enhancer locations than iRGCs (Figure 2C).

Consistent with the delayed birth and maturation of iRGCs compared with that of cRGCs, GO analysis of the accessible regions in iRGCs retrieved terms associated to eye morphogenesis, neural fate specification, and cell differentiation that were not detected in cRGCs (Figure 2D). Terms associated with axon guidance were retrieved in both populations. Interestingly, DARs located at putative enhancers (i.e., cis regulatory elements (CRE) upstream of the TSS and in intragenic regions) were associated with terms such as *A* *xon* *G* *uidance*, *A* *xon* *E* *xtension*, and *N* *euron* *P* *rojectio*n *G* *uidance* (Figure S2, Supporting Information). This observation suggested that axonal trajectory selection is highly influenced by the differential occupation of CREs rather than by regions located at the promoters.

### 2.3 Correlation Between Differential CRE Occupancy and Transcription

**Figure 3:**
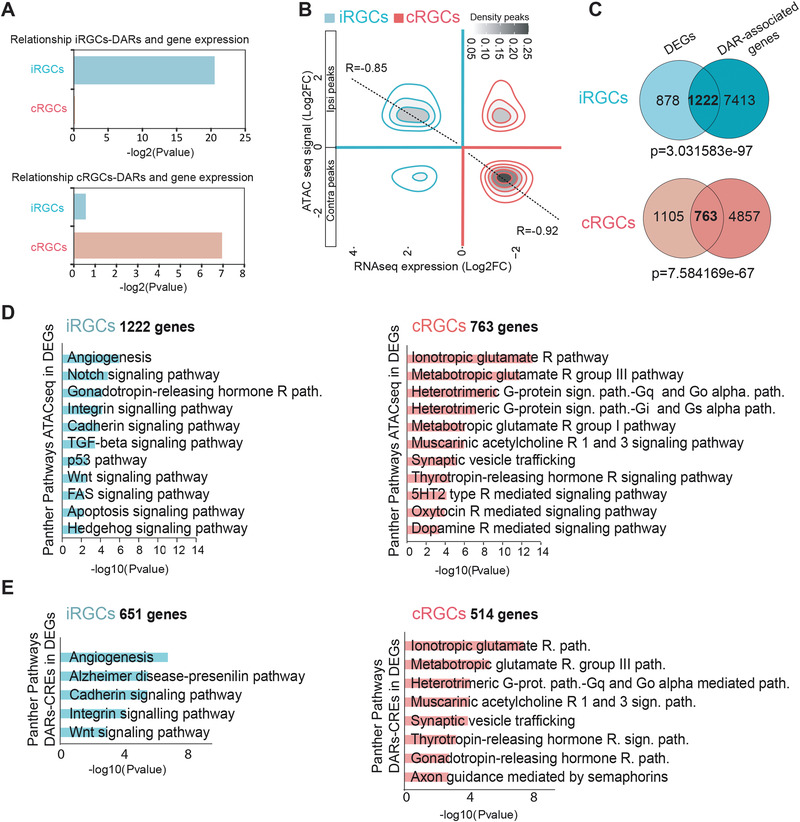
Transcriptomic and chromatin occupancy profiles define the set of genes differentially expressed in ipsilateral retinal ganglion cell (iRGC) and contralateral retinal ganglion cells (cRGCs). A) BETA P values confirm the association between differentially accessible regions (DARs) and gene expression (DEGs) in both iRGCs and cRGCs. B) Density plot showing chromatin accessibility regions and gene expression. Pearson correlation confirmed significant relationship between ATAC‐seq with RNA‐seq from iRGCs (*R* = 0.85) and ATAC‐seq combined with RNA‐seq for cRGCs (*R* = 0.92). C) Venn diagrams showing the overlap between peak‐associated genes from ATAC‐seq and DEGs from RNA‐Seq changes in iRGCs and cRGCs populations. Fisher's exact tests were used to evaluate if the number of ATAC‐seq peaks associated with genes was statistically significative. For ATAC‐seq peaks (Padj < 0.05 and |Log2FC|>1) we considered differential peaks between cRGC and iRGC that are associated with DEGs. Thus, there may be a number of peaks associated with the same gene. D) Panther pathways Gene Ontology (GO) enrichment analysis of DARs in cRGCs and iRGCs. Padj < 0.05 and |log2FC| ≥ 1. E) Panther pathways GO analysis of DEGs with DARs at cis regulatory elements (CREs). Padj < 0.05 and |log2FC| ≥ 1.

To determine the relationship between gene transcription and chromatine occupancy in the iRGC and cRGC populations, we next compared the ATAC‐seq and RNA‐seq datasets. Binding and expression target analysis (BETA)^[^ ^24^ ^]^ confirmed that the differences in chromatin accessibility were a good predictor of differential gene expression in both iRGCs and cRGCs (**Figure** **3**A). In addition, both iRGC‐DEGs and cRGC‐DEGs were compared with the iRGC‐DARs and cRGC‐DARs. We found a good correlation between the two signals in each cell subtype (Figure 3B). 58% of the DEGs in iRGCs and 40.8% of the DEGs in cRGCs were associated with DARs. Still, only 1,222 out of 3,968 (30,8%) DEGs displayed differential occupancy of the chromatin in iRGC and 763 (19,2%) in cRGCs (Figure 3C), indicating that other mechanisms also contribute to control the final levels of DEG transcripts.

PANTHER analysis of DEGs associated with iRGC‐DARs showed a high enrichment of the Wnt and Cadherin signaling pathways (Figure 3D), which agrees with recent studies showing that iRGCs require these pathways for midline avoidance.^[^ ^25^, ^26^ ^]^ These pathways were still retrieved when we restricted the PANTHER analysis to DEGs associated with DARs located at CREs (Figure 3E), further underscoring the relevance of CRE regulation in axon guidance.

### 2.4 Extended TF Code for Axon Midline Decisions

**Figure 4:**
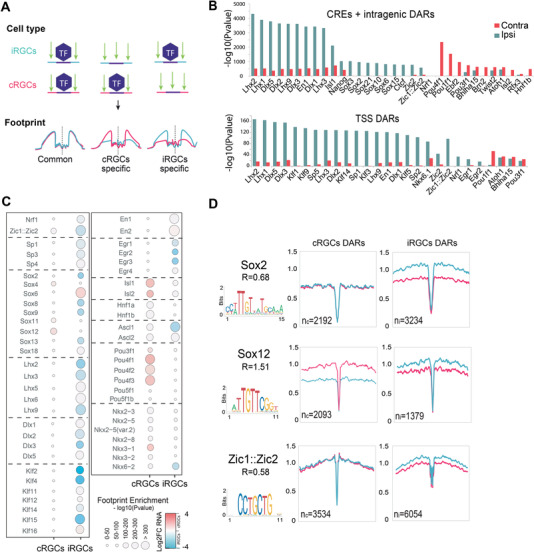
Transcription factor footprints in ipsilateral retinal ganglion cell (iRGC) and contralateral retinal ganglion cells (cRGCs). A) Diagram representation to illustrate the transcription factor (TF) binding enforcing characteristic patterns in the chromatin (footprint) of iRGCs and cRGCs. B) TF footprint enrichment at differential accessible regions (DARs) for cRGCs and iRGCs. We performed separated analysis in promoters (TSS) and putative enhancers (CRE) regions. C) Digital footprinting at enhancer/promoter sites indicating the motif enrichment (circle size) and the associated TF expression change in iRGC or cRGC RNA‐seq datasets. Red: upregulated; blue: downregulated; white: no‐change. D) Digital footprinting at enriched motifs specific for the indicated transcription factors. *n* = number of motifs detected in cRGCs (*n*_c_) or iRGCs (*n*_i_) (values correspond to normalized tn5 insertions). Motif matrix from JASPAR database is shown for each transcription factor. *R* = *n*_c_/*n*_i_

A very useful feature of ATAC‐seq is the analysis of TF footprints. The binding of TFs to DNA prevents Tn5 transposase cleavage within the binding site leading to a relative depletion of reads within the open chromatin region; as a result, actively bound TFs enforces characteristic patterns in the chromatin (**Figure** **4**A).^[^ ^27^ ^]^ To identify the members of the different families involved in axon guidance regulation, we searched TF footprints differentially detected in iRGC and cRGC‐specific DARs by using HINT (Hmm‐based Identification of Transcription factor footprints). TF motifs enrichment at footprinted sites revealed remarkable differences between cRGC and iRGC. The ipsilateral trajectory was associated with the presence of TFs belonging to the Lhx, Dlx, Sox, En, and Zic families at promoters and CREs. In contrast, cRGC‐specific DARs displayed a significant enrichment for other TFs such as Ebf1 or members of the Pou family (Figure 4B).

We next crossed the ATAC‐seq footprint analysis with the results from the RNA‐seq analysis to identify the members of the TF families that are differentially expressed in one or the other population, and therefore are more likely to be responsible for the differences observed in the digital footprints. These bioinformatical analysis retrieved families of TFs potentially involved in specifying the projection of contralateral (e.g., Pou3f and Pou4f, Ebf2) and ipsilateral neurons (e.g., Lhx, Dlx, Zic) thereby extending our knowledge of the transcriptional regulation of axon guidance. Intriguingly, some TF families occupy regulatory regions in both iRGCs and cRGCs depending on the member of the family. The most remarkable example is the Sox family, which has been involved in cRGCs guidance.^[^ ^28^ ^]^ While Sox2, Sox8, Sox9, and Sox13 were expressed in iRGC consistently with differences for the footprint of these TFs in iRGC chromatin, other members of the same family (e.g., Sox4 and Sox12) showed differential footprints and higher expression in cRGCs (Figure 4C,D). As a proof‐of‐principle to functionally validate our screen, next we focused our analysis in the Pou4f TFs for cRGCs and Zic2 and Lhx2/9 for iRGCs because they were retrieved in the footprint analysis and specifically expressed in cRGCs and iRGCs, respectively.

### 2.5 Pou4f1 Controls Midline Crossing and Regulates *γ*‐Synuclein

**Figure 5:**
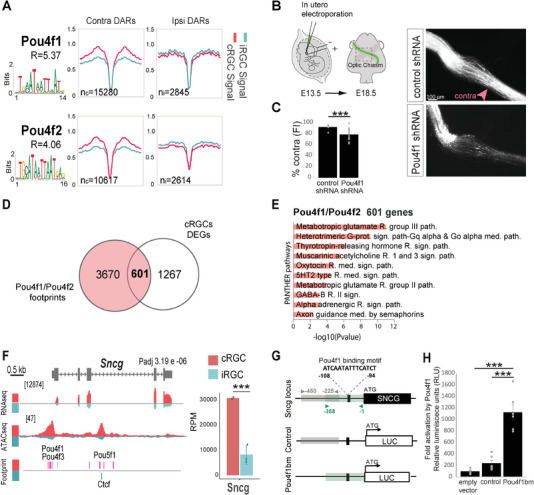
Pou4f1 controls midline crossing. A) Digital footprinting at enriched motifs in Pou4f1 and Pou4f2. *n* = number of motifs detected in contralateral retinal ganglion cells (cRGCs; *n*_c_) or ipsilateral retinal ganglion cells (iRGCs; *n*_i_) (values correspond to normalized tn5 insertions). Motif matrix from JASPAR database is shown for each transcription factor. *R* = *n*_c_/*n*_i_. B) Left panel is a schematic drawing of the experimental approach. Monocular in utero electroporation with plasmids encoding EGFP is performed in E13.5 embryos and their optic chiasms are analyzed at E18.5. Right panels despite optic chiasms of E18.5 embryos electroporated at E13.5 with plasmids encoding for scramble shRNA (control) or Pou4f1 shRNA plus EGFP encoding plasmids. Scale bar: 100 µm. C) Percentage of ipsilaterally projecting axons at the optic chiasm normalized to the total number of targeted axons (*n* = number of embryos; Control *n* = 6, Pou4f1 shRNA *n* = 16) (two‐tailed unpaired *t* test, ***p < 0.001). Results show means ± SEM. D) Venn diagram showing the overlap between differentially expressed genes (DEGs) in the cRGCs population and the footprints of Pou4f1 and Pou4f2 annotated to the nearest gene. E) Panther pathways Gene Ontology (GO) enrichment analysis of DEGs associated to the Pou4f1 and Pouf4f2 footprints in cRGCs. Padj < 0.1 and |log2FC| ≥ 1. F. Genomic snapshots of ATAC‐seq, RNA‐seq profiles and footprints in iRGCs and cRGCs populations for the *γ*‐synuclein encoding gene (Sncg). Notice that several footprints for Pou4f factors located upstream and inside *Sncg* are detected specifically for cRGCs. Values indicate the levels of counts in reads per million (RPM). Graphs represent transcripts expression (in RPM) for Sncg obtained from iRGC or cRGCs RNA‐seq experiments. (***Padj < 0.001). G) Schematic representation of Sncg gene including the Pou4f1 binding motif located in the promoter region of the gene, the region of 358 bp containing the Pou4f1 binding motif (pou4f1bm) amplified by PCR to be cloned in a luciferase reporter plasmid (pGL3‐basic) to generate the pGL3‐Sncg‐cre plasmid and the region of 225 bp next to the Sncg‐cre excluding the Pou4f1 motif amplified to be cloned into the pGL3 and used as a control (pGL3‐control). H) Luciferase assay in HEK293 cells showing a stronger transactivation of the reporter gene driven by Pou4f1 binding to its motif in Sncg gene respect to controls (*n* = number of samples; empty vector *n* = 6, control *n* = 6, pou4f1bm *n* = 6) (ANOVA test; followed by Bonferroni correction, ***p < 0.001).

Among the TFs with differential footprint occupancy and highly expressed in the cRGCs population we found Pou4f1 and Pou4f2 (**Figure** **5**A). In order to test a putative function of Pou4f TFs in axon midline crossing we performed LOF experiments. Plasmids encoding short hairpin RNAs against Pou4f1 (*Pou4f1shRNA*) or control plasmids (control shRNA) were delivered into the retina of E13.5 mouse embryos by in utero electroporation. The analysis of the electroporated embryos 4 days later, revealed that while RGC axons cross the midline in control embryos, embryos electroporated with *Pou4f1 shRNA* showed a reduced number of crossing axons. Also, a large number of axons stalled before entering the chiasm region (Figure 5B,C), demonstrating that Pou4f1 is essential for midline crossing as predicted by our computational analysis.

We then crossed the footprint analysis with the RNA‐seq datasets. This combination revealed that more than 30% of the genes differentially expressed (DEGs) in cRGCs showed footprints for Pou4f1 and/or Pou4f2 (Figure 5D). GO analysis of 593 DEGs with footprint for Pou4f1/2 retrieved terms related to axon guidance mediated by semaphorins (Figure 5E), in agreement with previous reports that demonstrate a role for Sema receptors in midline crossing.^[^ ^18^, ^29^ ^]^ Among the genes containing DARs and Pou4f1 footprints at regulatory regions we found *Sncg*, which encodes for a chaperon protein known as *γ*‐synuclein and according to this, the RNA‐seq profile of *Sncg* showed that *γ*‐synuclein mRNA is differentially expressed in cRGCs (Figure 5F), strongly suggesting that Pou4f1 regulates *γ*‐synuclein.

To investigate the potential capacity of Pou4f1 to activate *Sncg* transcription, a 358 bp sequence upstream of the *Sncg* transcription start site that contains the predicted Pou4f1 binding motif was amplified by PCR, cloned into a luciferase reporter plasmid and used for luciferase assays in 293HEK cells. A sequence of similar size also located upstream of the TSS but excluding the Pou4f1 motif was also cloned into the pGL3 and used as a control (Figure 5G). The transfection of the empty control plasmid plus the expression plasmid containing the Pou4f1 coding sequence resulted in basal luciferase activity. When the control plasmid bearing the control sequence was cotransfected with the pCDNA3‐Pou4f1 plasmid luciferase activity above the basal level was detected. The Pou4f1‐encoding plasmid transfected into 293HEK cells in combination with the plasmid that contained the predicted Pou4f1 motif lead to a more than 10‐fold increase in the luciferase levels compared to the basal levels (Figure 5H). This indicates that Pou4f1 can bind to this regulatory region at the *Sncg* locus and activate transcription.

**Figure 6:**
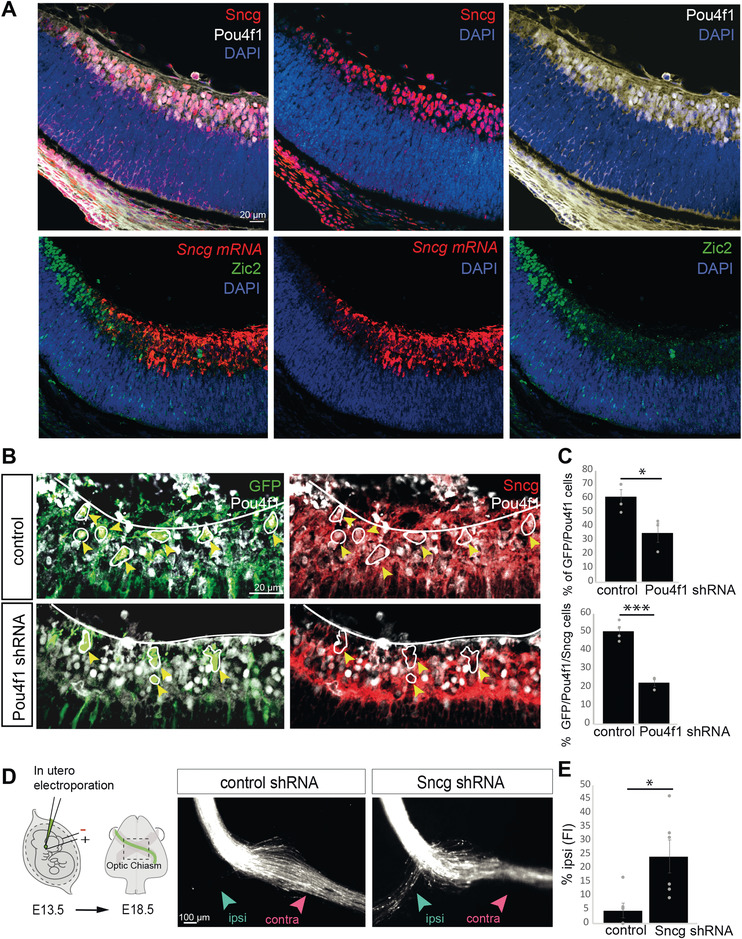
Pou4f1 regulation of *γ*‐synuclein. A) Immunofluorescence for Sncg combined with immunofluorescence for Pou4f1 (upper) or Zic2 (bottom) in coronal retinal sections from E16.5 embryos. Nuclei are counterstained with Dapi. Scale bar: 20 µm. B) Immunofluorescence for Sncg combined with immunofluorescence for Pou4f1 or GFP in coronal retinal sections from E18.5 embryos after retina electroporation with plasmids encoding for scramble shRNA (control) (upper panels) or *Pou4f1* shRNA (bottom panels) plus EGFP encoding plasmids. Scale bar: 20 µm. C) Quantification of the percentage of GFP/Pou4f1 positive cells and GFP/Pou4f1/Sncg positive cells in retinal sections from E18.5 embryos after retina electroporation with plasmids encoding for scramble shRNA (control) or *Pou4f1* shRNA plus EGFP encoding plasmids (*n* = number of embryos; Control *n* = 4, Pou4f1 shRNA *n* = 3) (at least three sections for animal) (two‐tailed unpaired *t* test, *p < 0.05, ***p < 0.001). Results show means ± SEM. D) Left panel is a schematic drawing of the experimental approach. Monocular in utero electroporation with plasmids encoding EGFP is performed in E13.5 embryos and their optic chiasms are analyzed at E18.5. Right panels despite optic chiasms of E18.5 embryos electroporated at E13.5 with plasmids encoding for scramble shRNA (control) or *Sncg* shRNA plus EGFP encoding plasmids. Scale bar: 100 µm. E) Percentage of ipsilaterally projecting axons at the optic chiasm normalized to the total number of targeted axons (*n* = number of embryos; Control *n* = 6, Sncg shRNA *n* = 6) (two‐tailed unpaired *t* test, *p < 0.05). Results show means ± SEM.

To investigate if Pou4f1 activates *γ*‐synuclein in cRGCs in vivo, we first performed triple immunolabeling for *γ*‐synuclein, Pou4f1 and Zic2 in retinal sections from E16.5 embryos and confirmed the selective expression of *γ*‐synuclein in cRGC, its colocalization with Pou4f1, and its complementary pattern with Zic2 (**Figure** **6**A). Then, we analyzed the expression of *γ*‐synuclein after downregulation of Pou4f1 by electroporating Pou4f1 shRNA into the retina of E13.5 embryos and performed *γ*‐synuclein immunostaining in E16.5 retinal sections. We observed that 61.5% of the cells expressed Pou4f1 and GFP in the RGC layer and 50.5% were also positive for *γ*‐synuclein. However, the number of GFP cells that expressed Pou4f1 and *γ*‐synuclein was significantly reduced (22.6%) in the retinas of *Pou4f1 shRNA*‐electroporated embryos (Figure 6B,C), demonstrating that the expression of *γ*‐synuclein in cRGCs depends on Pou4f1.

The Synuclein family is associated to pathogenesis of neurodegenerative diseases and tumor development.^[^ ^30^, ^31^, ^32^ ^]^ *γ*‐synuclein predominantly localizes in axons and presynaptic terminals,^[^ ^33^ ^]^ and a recent study has shown that long‐term exposure of young neurons to *α*‐synuclein, another member of the same family, hampers axon elongation and growth cone turning.^[^ ^34^ ^]^ However, a role for *γ*‐synuclein in neural development has not been previously reported. Therefore, we investigated *γ*‐synuclein function in RGCs by performing LOF experiments delivering plasmids encoding short hairpin RNAs against Sncg (*Sncg shRNA*) into the retina of E13.5 mouse embryos (Figure 6D). The analysis of RGCs axonal trajectories at the optic chiasm level 4 days after electroporation, showed a significant increase in the number of axons switching their laterality at the midline (Figure 6E). Together all these results indicate that *γ*‐synuclein is expressed in cRGCs, participates in midline crossing and is a Pou4f1 target.

### 2.6 Zic2‐Mediated Regulation of Axon Midline Avoidance

**Figure 7:**
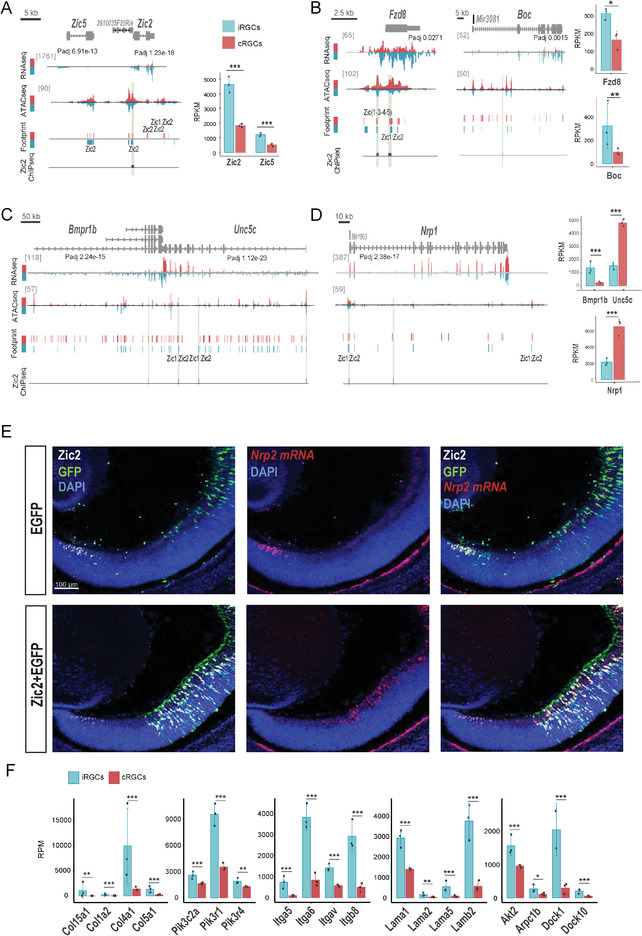
Known and novel differentially expressed genes (DEGs) in ipsilateral retinal ganglion cell (iRGC) or contralateral retinal ganglion cells (cRGCs). A) Genomic snapshots of RNA‐seq, ATAC‐seq, digital footprints, and Zic2 ChIP‐seq in iRGC and cRGCs at the *Zic2/Zic5* locus. Zic2 transcripts are highly expressed in iRGCs. The Zic2/Zic5 locus contains several potential Zic1:Zic2 footprints. Actual binding of Zic2 to the site located in the TSS of Zic2 was confirmed by ChIP‐Seq (green band). Graph at the right represents transcripts expression for Zic2 and Zic5 (in reads per million, RPM) obtained from iRGC or cRGCs RNA‐seq experiments. B–D) Genomic snapshots of RNA‐seq, ATAC‐seq, digital footprints in cRGC (red)/iRGCs (blue), and Zic2 ChIP‐seq in retina/spinal cord at loci that encode for receptors differentially expressed in iRGCs and cRGCs. Most of these loci contain potential Zic1:Zic2 footprints and some of them were confirmed by ChIP‐seq (green band). E) Coronal retinal sections from E16.5 embryos electroporated at E13.5 with plasmids encoding for EGFP‐control plasmids (upper panels) or Zic2‐ plus EGFP‐encoding plasmids (bottom panels). Immunostaining for Zic2 combined with in situ hybridization for *Nrp2 mRNA* demonstrate that Nrp2 is highly and specifically expressed in endogenous Zic2 positive cells in the peripheral retina but not in targeted cells expressing EGFP. However, when Zic2 is ectopically expressed, *Nrp2 mRNA* levels are highly increased. Scale bar: 100 µm. F) Graphs represent transcripts expression (in RPM) obtained from iRGC or cRGCs RNA‐seq experiments corresponding to genes encoding for different integrins (‐*t* test, ***Padj < 0.001).

Previous studies have identified Zic2 as a key regulator of axon midline avoidance, first in the visual system and later in other neuronal types including spinal and thalamocortical neurons.^[^ ^21^, ^35^, ^36^ ^]^ Consistent with these studies, Zic2 showed differential DARs and expression in iRGCs versus cRGCs (Figure 4C,D and **Figure** **7**A). In agreement with a recent scRNA‐seq analysis of E15.5 retinas,^[^ ^37^ ^]^ we also detected differential expression of Zic5, which is located next to Zic2 in an inverted orientation (Figure 7A) in the RNA‐seq analysis of iRGC versus cRGCs. However, the level of Zic5 transcripts abundance was likely neglectable compared to *Zic2* transcripts (Figure 7A).

Taking advantage of a published Zic2 ChIP‐seq analysis carried out in E16 retinas and spinal cords^[^ ^26^ ^]^ we next compared digital Zic2 footprints obtained from the ATAC‐seq data with actual Zic2 binding across the genome (see the Experimental Section). About 7% of the Zic2 footprints (ATAC‐seq) retrieved using HINT, were occupied by Zic2 (ChIP‐Seq) in iRGCs. Interestingly, HINT analysis predicted two Zic2 footprints between the *Zic2* and *Zic5* loci and the ChIP‐Seq data demonstrated actual Zic2 binding to the DAR closer to *Zic2* (Figure 7A). This binding of Zic2 to a genomic region close to the Zic2 locus suggests that Zic2 may be regulated by a feedback or feedforward mechanism.

The combination of transcriptomic, ChIP‐seq, and chromatin occupancy data also retrieved many axon guidance receptors encoding for proteins previously implicated in axon pathfinding such as Fzd8, Boc and Bmpr1.^[^ ^17^, ^26^, ^38^ ^]^ In line with these reports, we found differential expression of *Fzd8, Boc*, and *Bmpr1* in iRGCs (Figure 7B,C) and detected differentially occupied CREs with predicted Zic2 footprints in the *Fzd8* and *Bmpr1* loci (Figure 7B,C). Some of these predicted Zic2 footprints coincided with actual Zic2 binding, in agreement with previous reports demonstrating that these two genes are regulated by Zic2.^[^ ^26^, ^38^ ^]^ In the case of Boc, we observed actual Zic2 binding inside the gene suggesting that this receptor may be also regulated by Zic2 (Figure 7B,C). Unc5c, a Netrin1 receptor negatively regulated by Zic2^[^ ^16^ ^]^ also showed Zic2 binding in several regions across its locus (Figure 7C) suggesting that Zic2 binding to this region acts as a repressor. Interestingly, Unc5c and Bmpr1b are located next to each other in inverted directions (Figure 7C). According to the footprint analysis, the region that coincides with the beginning of *Unc5c* and the end of *Bpm1r* contains Zic2 binding motifs and indeed actual Zic2 binding was found in these regions, suggesting that *Unc5c* and *Bmp1r* may be simultaneously regulated by Zic2.

We also found Zic2 binding close to the Semaphorin receptor, Nrp1, previously reported as expressed in cRGC and not in iRGCs.^[^ ^39^ ^]^ As in the case of Unc5c, these results suggested that Zic2 may negatively regulate Nrp1 in iRGCs. In addition, we identified several receptors potentially enriched in iRGCs not previously reported as specific of this neuronal population. For instance, the *Nrp1* paralog *Nrp2* contains DARs and we detected actual Zic2 binding at the promoter region of this gene. In agreement with a potential role for Zic2 in regulating *Nrp2* expression, Zic2 and *Nrp2* mRNA colocalized in the ventral retina of E16 embryos (Figure 7E). Moreover, ectopic expression of Zic2 in cRGCs by in utero electroporation led to a significant increase of *Nrp2* mRNA levels in the same cells (Figure 7E), experimentally confirming Nrp2 as a target of Zic2. *Nrp2* has been proposed to promote *α*5‐Integrin (aka Itga5) recycling in migrating endothelial cells during angiogenesis.^[^ ^40^ ^]^ Interestingly, our PANTHER analysis of DEGs associated with iRGC‐DARs data retrieved Integrin signaling as one of the most enriched terms in iRGCs. In line with these data, a recent study suggested that integrins are essential to restrict iRGCs targeting to the sublamina of the superior colliculus^[^ ^41^ ^]^ (Figure 7F). We found Itga5 as well as many other integrins specifically expressed in iRGCs and most of them present binding of Zic2 in their genomic sequences (Figure S3, Supporting Information). All together, these data strongly suggest that Zic2 regulates the expression of many members of this family of adhesion proteins in iRGCs.

In conclusion, in addition to retrieve many genes previously implicated in axon guidance at the chiasm, our screen led to the unbiased identification of new Zic2 targets potentially involved in this developmental process.

### 2.7 Lhx2/9 Coregulate with Zic2 the Ipsilateral Trajectory

**Figure 8:**
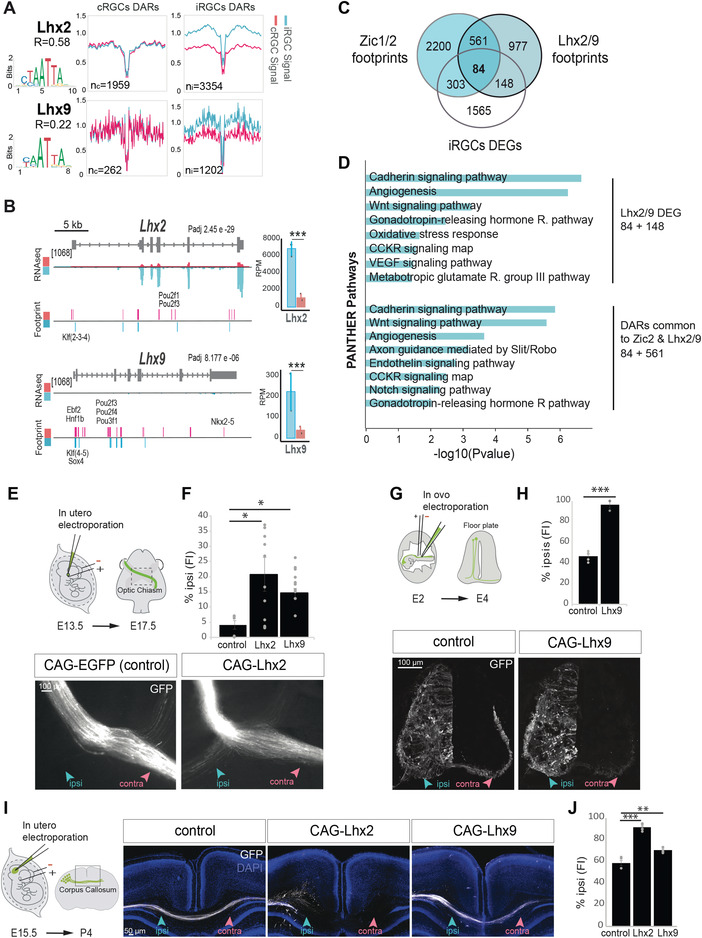
Lhx2/9 as regulators of axon midline avoidance. A) Digital footprinting at Lhx2 and Lhx9 motifs. *n* = number of motifs detected in contralateral retinal ganglion cells (cRGCs; *n*_c_) or ipsilateral retinal ganglion cells (iRGCs; *n*_i_) (values correspond to normalized tn5 insertions). Motif matrix from JASPAR database is shown for each transcription factor. *R* = *n*_c_/*n*_i._ B) Genomic snapshots of ATAC‐seq, RNA‐seq profiles, and footprint enrichment in iRGCs and cRGCs at the *Lhx2* and *Lhx9* loci represented in reads per million (RPM). ***Padj < 0.001 Graphs represent transcripts expression (in RPM) for Lhx2 and Lhx9 obtained from iRGC or cRGCs RNA‐seq experiments. C) Venn diagram showing the overlap between the differentially expressed genes (DEGs) and footprints for Zic1/2 and Lhx2/9 annotated to the nearest gene in the iRGCs population. D) Panther pathways Gene Ontology (GO) enrichment analysis of DEGs associated to the Lhx2/9 footprints in iRGCs, and common to Zic1/2 and Lhx2/9. Padj < 0.1 and |log2FC| ≥ 1. E. Upper is a schematic drawing of the experimental approach. Monocular in utero electroporation with plasmids encoding EGFP is performed in E13.5 embryos and their optic chiasms are analyzed at E18.5. Optic chiasms of E18.5 embryos monocularly electroporated at E13.5 with plasmids encoding for the transcription factor Lhx2 and EGFP or EGFP alone as a control. Embryos electroporated with Lhx2 displayed an ectopic ipsilateral projection not present in the controls. Scale bar: 100 µm. F) The graph represents the percentage of ipsilaterally projecting axons at the optic chiasm normalized to the total number of targeted axons in embryos electroporated with Lhx2, Lhx9, and/or EGFP encoding plasmids (*n* = number of embryos; control *n* = 6, Lhx2 *n* = 12, Lhx9 *n* = 11) (ANOVA test; followed by Bonferroni correction, *p < 0.05). Results show means ± SEM. G) Upper panel is a schematic drawing of the experimental approach. Transverse sections of spinal cords from E4 chick embryos unilaterally electroporated at E2 with plasmids encoding for Lhx9 and/or EGFP. H) The graph represents the percentage of ipsilaterally projecting axons normalized to the total number of targeted axons (*n* = number of embryos; control *n* = 5, Lhx9 *n* = 3) (two‐tailed unpaired *t* test, ***p < 0.001). Results show means ± SEM. I) Left panel is a schematic drawing of the experimental approach. Coronal sections from P4 brains of mice electroporated at e15.5 with plasmids encoding EGFP and/or Lhx2 or Lhx9. J. The graph shows the percentage of ipsilaterally projecting axons normalized to the total number of targeted axons (*n* = number of pups; Control *n* = 4, Lhx2 = 5, Lhx9 *n* = 5) (ANOVA test; followed by Bonferroni correction, **p < 0.01, ***p < 0.001). Results show means ± SEM.

In addition to the expected differential occupancy of Zic2 footprints, our ATAC‐seq screen retrieved differential occupancy of Lhx2/9 binding motifs in iRGCs (**Figure** **8**A). Lhx2 and Lhx9 are both expressed in the developing retina^[^ ^42^, ^43^ ^]^ (Figure 8B) but it is unknown whether they contribute to the wiring of RGC axons. Lhx2 zebrafish mutants show disrupted crossing at the chiasmatic midline although this defect has been described as secondary due to a disruption in the patterning of the midline.^[^ ^44^, ^45^ ^]^ Thus, we decided to investigate in more detail the role of Lhx2/9 in the guidance of iRGC axons. Lhx9 transcripts levels are higher in iRGCs than in cRGCs albeit its expression is low in both populations. In contrast, Lhx2 transcripts are highly expressed in iRGCs (Figure 8B).

To study a putative role for Lhx2/9 in axon guidance at the optic chiasm, we performed gain‐of‐function (GOF) experiments by ectopically expressing Lhx2 or Lhx9 in cRGCs. For that, E13.5 mouse embryos were monocularly injected with Lhx2 or Lhx9‐encoding plasmids together with GFP‐plasmids and electroporated in utero. The axons of targeted cRGCs were examined 4 days later. A significant increase in the number of axons projecting ipsilaterally was clearly observed after ectopic expression of either Lhx2 or Lhx9 (Figure 8E,F), indicating that these transcription factors influence axon guidance by promoting ipsilaterality. These results suggest that the occupancy of Lhx2/9‐CRE is key in the regulation of axon guidance, possibly through the control of the Wnt pathway. Note that although Lhx2 and Lhx9 may be interchangeable in their function as axon guidance regulators, Lhx2 is likely to play a more prominent role in RGCs given its higher expression level in those cells (Figure 8B).

Because these TFs are specifically expressed in iRGCs, we wondered if their targets overlapped with the program potentially regulated by Zic2. To address this question, we analyzed the list of genes associated with Lhx2/9 and Zic1/2 footprints in iRGCs and found that 36.5% of the genes containing Lhx2/9 footprints could be also regulated by Zic1/2 (645 out of 1770) (Figure 8C). GO analysis of DEGs potentially regulated by Lhx2/9 (84+148=232) retrieved a large enrichment in genes related to the cadherin and Wnt pathways, two pathways previously linked to Zic2^[^ ^26^ ^]^ (Figure 8D). These results indicate that Lhx2/9 and Zic2 may regulate a common set of genes during the guidance of RGC axons to the brain.

Finally, because Lhx TFs are expressed in other contexts during development we investigated their participation in axon guidance in other circuits. Previous studies have shown that Lhx2 and Lhx9 are both expressed in the dl1 interneuron population during spinal cord development and mice lacking both TFs exhibit midline crossing defects at the floor plate (Wilson et al., 2008). The dl1 spinal population contains both commissural (dl1_c_) and ipsilateral (dl1_i_) spinal neurons. *Lhx2* and *Lhx9* are both highly expressed in recently differentiated dl1, but are later specifically downregulated in dl1_c_ (more precisely, *Lhx2* disappears from dl1_i_, while *Lhx9* is maintained at high levels in the dl1_i_ and low levels in the dl1_c_ population). We performed Lhx9 GOF by *in ovo* electroporation into the chicken spinal cord and found a significant increase in the number of ipsilaterally projecting axons concomitant with a strong reduction of commissural axons (Figure 8G,H). This result confirms that Lhx2/9 factors are implicated in defining the laterality of spinal axons and reveals that, like Zic2,^[^ ^35^ ^]^ Lhx9 is sufficient to induce axon midline avoidance not only in the retina but also in the spinal cord.

Lhx2/9 factors are also expressed in the developing cortex regulating the specification of cortical neurons.^[^ ^46^, ^47^ ^]^ It is, however, unknown whether they play a role in the guidance of cortical axons. To assess this possibility, we electroporated Lhx2 or Lhx9‐encoding plasmids plus GFP plasmids into the telencephalon of E15 embryos and analyzed the projections of targeted cortical neurons at P4. Axons from cortical neurons ectopically expressing either Lhx2 or Lhx9 avoided the midline at the corpus callosum. Similar to the results observed in the visual system, the phenotype induced by Lhx2 was significantly stronger than the one triggered by Lhx9 (Figure 8I,J).

Together, these data indicate that Lhx2/9 regulate axon trajectories in different systems, although the specific member of the family that regulate the process may differ between circuits. In addition, we observed an intriguing convergence of Lhx and Zic2 functions which agrees with the large number of common targets between these TFs.

## 3 Discussion

Here, we profiled in parallel the transcriptome and the accessibility and occupancy of chromatin in two neuronal populations with similar functions but divergent projection behaviors. This strategy allowed us to identify a number of genes potentially involved in the regulation of axon trajectories during development, opening new lines of research. Our analyses also provided molecular insight into the general mechanisms regulating axon guidance decisions in the visual circuit. For instance, the footprint analysis revealed that most of the retrieved DEGs related to axon guidance were associated with DARs located at CRE rather than promoters, underscoring an unexpected contribution of differential enhancers used in axon guidance.

Our screen retrieved the majority of receptors and intracellular proteins previously implicated in the navigation of visual axons at the midline including genes previously described elsewhere^[^ ^48^ ^]^ and demonstrated the differential binding of various TFs involved in the selection of axonal trajectories. It also identified novel guidance receptors, such as Nrp2, implicated in axon guidance at the midline. The identification of novel TFs and downstream targets brings us a step closer to a complete inventory of factors controlling axon guidance at the midline.

The formation of the optic chiasm in mouse occurs in two sequential steps. In a first phase, pioneer axons arising from dorso‐central retina leave the optic cup through the optic disc at E12 and navigate into the optic stalk to enter the developing ventral diencephalon. The majority of these pioneering axons then cross the midline and grow in close relationship with an inverted V‐shaped^[^ ^49^ ^]^ array of early neurons, establishing the position of the optic chiasm along the anterior–posterior axis of the brain. A small proportion of these axons extend on the same side of the brain, distant from the midline, and form an early and transient uncrossed projection that abruptly disappear after E16.^[^ ^50^, ^51^, ^52^ ^]^ It is known that these transient RGCs growing into the ipsilateral side do not express Zic2 or Slc6a4.^[^ ^12^, ^21^, ^52^ ^]^ Since our screen is performed at E16, it remains unclear whether this transient ipsilateral population, that is considered the product of guidance errors, express other typical ipsilateral markers.

Recent studies using single‐cell RNA‐seq to profile RGCs from embryonic and postnatal animals have demonstrated that subtype diversification arises as a gradual, asynchronous fate restriction of postmitotic multipotential precursors and some types of neurons are not identifiable until sometime after their differentiation.^[^ ^53^ ^]^ In this study, the authors suggest that axonal laterality is specified before type identity is completely fixed and also that immature RGCs are multipotential even after selecting the ipsi or the contralateral trajectory.^[^ ^53^ ^]^ Our results support this view and suggest that recently differentiated RGCs are doubly specified by laterality and type but once axonal choice has been made the laterality program is shut down.

To validate our bioinformatic analysis, we explored in greater detail two of the most interesting TF families retrieved in our screen. In particular, we provided further information about the role of Pou4f and Lhx TFs in axon guidance. Pou4f1 has been referred to as a specific marker of cRGCs,^[^ ^54^ ^]^ however, no role in axon guidance has been reported for this TF. We discovered that Pou4f1 is essential for midline crossing, is able to activate transcription and essential for the expression of *γ*‐synuclein. The synuclein family consists of three members *α*‐synuclein, *β*‐synuclein and *γ*‐synuclein. We found that *γ*‐synuclein, but no other members of the family, is differentially expressed in cRGCs and both transcriptomics and chromatin occupancy profiles pinpoint *γ*‐synuclein as the only member of the family playing a role in the guidance of visual axons. Although it was known that *γ*‐synuclein is expressed in adult RGCs,^[^ ^55^ ^]^ a role for this protein or any other member of the family in neural development had not been described. In vitro assays in HeLa cells revealed that *γ*‐synuclein can bind and promote tubulin polymerization, induce the microtubule bundling, and alter microtubules morphology through the microtubule‐binding protein MAP1. However, alterations of *γ*‐synuclein in HEK cells did not lead to any prominent phenotype.^[^ ^56^ ^]^ These observations may support the idea that the binding of *γ*‐synuclein to microtubule‐regulatory proteins is specific for its function in axonal navigation and future functional assays in RGCs should tackle the postulated link between *γ*‐synuclein and microtubule polymerization in vivo. The phenotypes observed after Pou4f1 and *γ*‐synuclein downregulation were not exactly alike. While Pou4f1 loss‐of‐function lead to axon stalling at the chiasm, the negative regulation of *γ*‐synuclein induced an ectopic ipsilateral projection. The fact that Pou4f1 LOF produces a stronger phenotype than *γ*‐synuclein downregulation might reflect the finding that *γ*‐synuclein is a Pou4f1 target.

Lhx factors play important roles in cortical fate determination and had been involved in the guidance of spinal axons.^[^ ^46^, ^47^, ^57^ ^]^ However, their role in the guidance of visual axons and other neuronal types had not been investigated. Our results confirmed the role of Lhx TFs in axon guidance decisions in different contexts. We observed that both Lhx2 and Lhx9 induce axon midline turning in retinal and cortical neurons, although Lhx2 appears to be more efficient triggering the midline avoidance program in these two cell types. However, the situation appears to be different in the spinal cord where Lhx9 is highly expressed in ipsilaterally projecting neurons and prevents midline crossing. Thus, it seems likely that Lhx2/9 bind to the CRE of different guidance molecules depending on the cell type. In line with this idea, it has been reported that while Lhx2 regulates Robo3 in dl1_c_ neurons, it seems to repress a different member of the Robo family, Robo1 in thalamocortical neurons.^[^ ^58^ ^]^ In zebrafish, Lhx2/9 negatively regulates the expression of Slits and Sema3a in the diencephalon^[^ ^44^ ^]^ again arguing cell‐type differences in the set of guidance molecules regulated by Lhx TFs.

In summary, our analyses have led to the identification of dozens of new genes potentially involved in axonal trajectory selection. These results open the door for innovative therapeutic approaches aimed to restore damaged neuronal circuits that will benefit from a better understanding of the mechanisms driving and constraining neuronal circuits assembly.

## 4 Experimental Section

### Animals

The ET33 Sert‐Cre line was generated by GENSAT^[^ ^59^ ^]^ and obtained from the MMRRC (http://www.mmrrc.org/strains/17260/017260.html).

The Pou4f2‐Cre mouse line generously donated by Dr. George Vann Bennet, Duke University Medical Center, was generated by gene targeting into the Brn3b locus.^[^ ^60^ ^]^ The Sun1‐tagged mice (Sun1GFP^f^) were acquired from Jackson Laboratories (Stock number 021039). These mice contain a CAG promoter driving expression of coding sequences for the mouse nuclear membrane protein Sun1 (Sad1 and UNC84 domain containing 1) fused at its C‐terminus to 2 copies of superfolder GFP (sfGFP) followed by six copies of Myc, inserted in the Gt(ROSA)26Sor locus. Expression of Sun1‐sfGFP‐Myc is prevented by an upstream loxP‐flanked stop cassette (Mo et al; Fernandez‐Albert et al). Cre‐dependent removal of the loxP‐STOP‐loxP cassette allows expression of the Sun1 fusion protein at the inner nuclear membrane in targeted cell types. These nuclei can be immunopurified with antibodies against GFP or MYC for transcriptional and epigenetic studies. All the electroporation experiments were performed using embryos from C57/DBA F1 hybrids. Mice were kept in a timed pregnancy breeding colony at the Instituto de Neurociencias (IN). Fertilized chicken eggs were obtained from a local farm (Granja Santa Isabel, Córdoba, Spain). The animal protocols were approved by the regional IN‐Animal Care and Use Committee (Consellería d'agricultura, desenvolupament rural, emergencia climatica I transicio ecologica, Ref Number: 2021/VSC/PEA/0210) met European and Spanish regulations.

### RNA‐seq and Analysis

Retinas from embryos at E16.5 were dissected in HBSS buffer without Ca^2+^ and Mg^2+^. In the case of retinas from Pou4f2^SunGFP^ embryos the ventrotemporal region was removed by dissection. Retinas were digested and disaggregated with the help of a P1000 using an enzymatic solution composed by colagenase, trypsin 0125%, BSA 0.2 mg mL^−1^ and DNAse 1 µL/mL^−1^ during 25′ at 37 °C. Digestion was stopped by adding DMEM with fetal bovine serum at 10% and disaggregation of the tissue was checked under microscope. The suspension was filtered and centrifugated at 1250 rpm during 5′ at RT. The pellet was resuspended and sorted by flow cytometry to harvest the interest cells. RNA was extracted from sorted cells using PicoPure RNA Isolation Kit (Arcturus) and libraries were made and sequenced in a HiSeq 2500 sequencer (Illumina, Inc). Reads were mapped using default parameters from HISAT2 v2.1.0 to the mouse genome (mm10). Mapped reads were annotated to genes from Ensemble (GRCm38.89) and quantified using HTseq v0.11.1 to enumerate the number of reads per sample. Differential expression analysis between tissue types was conducted with DEseq2 v1.26.0. in R and FDR was set to 10% (BH correction). GO enrichment analyses were performed using the platform PANTHER with Fisher's exact test and with the Padj correction, obtaining the top terms using the filters by ratio enrichment of >2, number of GO family group genes between 3 and 2000, number of enrichment genes of >3, and Padj < 0.1. Deeptools bamCoverage (v3.1.3, duplicate reads ignored, RPKM normalized and extended reads) was used to generate bigwig files from sorted and indexed bam files. RNA‐seq samples tracks were visualized using IGV (v2.3.72). Further data processing was performed with custom scripts in the R programming language (“Planting of a Tree”) (v3.6.1) and bioconductor v3.10.

Reads (fastq)   Read length    Mapped reads (Bamfiles)
Sample1_Slc6a4   42612648   50     36353413 (85.31%)
Sample2_Slc6a4   44101908   50     37853017 (85.83%)
Sample3_Slc6a4   42016591   50     35619716 (84.78%)
Sample4_Pou4f2   42418866   50    37254672 (87.83%)
Sample5_Pou4f2   43345470   50    37726956 (87.04%)
Sample6_Pou4f2   47918156   50    41730677 (87.09%)

### ATAC‐seq and Analysis

ATAC‐seq was performed in biological replicates (*n* = 3) and each replicate contained 50 000 cells from 8–10 embryos for each contralateral sample and from 30–40 embryos for each ipsilateral sample. Retinas from embryos at E16.5 were dissected in HBSS buffer without Ca^2+^ and Mg^2+^. In the case of retinas from Pou4f2^SunGFP^ embryos the ventrotemporal region was removed by dissection. Retinas were digested and disaggregated with the help of a P1000 using an enzymatic solution composed by colagenase, Trypsin 0125%, BSA 0.2 mg mL^−1^ and DNAse 1 µL mL^−1^ during 25 min at 37 °C. Digestion was stopped by adding DMEM with fetal bovine serum at 10% and disaggregation of the tissue was checked under microscope. The suspension was filtered and centrifuged at 1250 rpm during 5 min at RT. The pellet was resuspended and sorted by flow cytometry to harvest 50 000 green fluorescent cells. Cell suspension was spin down at 500 g for 5 min at 4 °C and treated with cold lysis buffer (10 × 10^−3^ m TrisCl ph7.4, 10 × 10^−3^ m NaCl, 3 × 10^−3^ m MgCl_2_, 0.1% Igepal). The suspension was centrifuged at 500 g during 10 min at 4 °C. The supernatant was discarded, and was make sure that the pellet was placed on ice. After that the transcription reaction and purification was continued following the protocol.^[^ ^22^, ^61^ ^]^ Then, the libraries were purified and sequenced by an Illumina HiSeq 2500 sequencer (Illumina, Inc). Paired‐end reads were aligned with Bowtie2 v2.3.4.2 to mm10 mouse genome. Duplicated reads were removed with Picardtools v2.18.21. MAPQ > 20 was used to filter nonunique alignments. BAM files for bulk ATAC‐seq were downsampled to a fixed number of reads using SAMtools v.1.9. Peak calling was performed with MACS2 v2.1.1 as indicated in.^[^ ^23^ ^]^ DARs analysis was performed using DiffBind v2.6.6. Regions with FDR < 0.05 and |log2FC| > 1 were considered significantly regulated. DARs and footprints were annotated to closest genes from Ensembl (GRCm38.89) using the Bioconductor package ChIPpeakAnno v3.20.1. Predictive relationship of DARs to gene expression changes was performed using BETA v1.0.7 using as reference genes from Ensembl (GRCm38.89). Pearson's correlation is the ratio between the covariance of two variables and the product of their standard deviations, this coefficient was used to calculate the correlation between ATACseq signal and RNAseq signal for DARs. Fisher's exact tests that are based on the hypergeometric distribution, were used to test if the genes associated with peaks of ATAC‐seq were statistically significative associated with gene expression.

Motif analysis of ATAC regions was performed using MEME‐suite v4.12.0. and JASPAR 2020 database. For digital footprint, the ATAC‐seq dedicated software HINT v0.12.3 (Hmm‐based IdeNtification of Transcription factor footprints) v0.12.3 was used and to discover enrichment of predicted DNA binding motifs for further analysis, the Homer function findMotifsGenome.pl using suggested basic usage settings HOMER v4.11 was employed. The bedtools genomecov function (v2.26.0) was then used to convert the list of locations into a genome coverage track containing the ATAC‐seq signal at each genomic position. Deeptools bamCoverage (v3.1.3, duplicate reads ignored, RPKM normalized and extended reads) was used to generate bigwig files from sorted and indexed bam files. ATAC‐seq samples tracks were visualized using IGV (v2.3.72). Further data processing was performed with custom scripts in the R programming language (“Planting of a Tree”) (v3.6.1) and bioconductor v3.10.

### RNA‐seq, ATAC‐seq, and ChIP‐seq Integration

Raw ChIP‐seq sequencing data from (Morenilla et al. 2020) were aligned to the mouse genome using Bowtie2. Based on these aligned reads, immuno‐enriched areas using the peak caller MACS2 were found. To integrate RNA‐seq, ATAC‐seq and ChIP‐seq, bedtools were used to overlap genomic intervals and visualize the alignment on the IGV/UCSC browser in specific loci.

### In Utero and In Ovo Electroporation

Lhx2 and Lhx9 coding sequences were cloned in the mammalian expression plasmid pCAG. Mouse overexpression target sequence was cloned using the following primers for Lhx9: Fw 5′CCTTGGGTACCACCATGGAAATAGTGGGGTGCCGAGC 3′ and Rv

5′CTTAGCCTCGAGTTAGGGAATTTTCAAACGTCGGGA 3′ and the following primers for Lhx2: Fw 5′CCTTGGGTACCGATGCACTGGGCCGGTTA 3′ and Rv 5′CTTAGCGAATTCAAAGAAATCGTGGGGGCTCG 3′. Pou4f1 shRNA and Sncg shRNA target sequence were designed using the GenScript siRNA Target Finder tool located at https://www.genscript.com/ssl‐bin/app/rnai and cloned into the pSilencer2.1 plasmid using the pSilencer Kit (Thermo Fisher Scientific) in accordance with the manufacturer's recommendations. Mouse Pou4f1 shRNA target sequence was cloned using the following primers: Fw 5′ GATCCGCCACGTACCACACGATGAATTTCAAGAGAATTCATCGTGTGGTACGTGGCTTTTTTGGAAA and Rv 5′ AGCTTTTCCAAAAAAGCCACGTACCACACGATGAATTCTCTTGAAATTCATCGTGTGGTACGTGGCG. Mouse Sncg shRNA target sequence was cloned using the following primers: Fw 5′ GATCCGCCAAGAGTGGAGAAGACTTTCAAGAGAAGTCTTCTCCACTCTTGGCTTTTTTGGAAA 3′ and Rv 5′ AGCTTTTCCAAAAAAGCCAAGAGTGGAGAAGACTTCTCTTGAAAGTCTTCTCCACTCTTGGCG 3′. Plasmidic DNA solution was injected into embryonic retinas or ventricles as described previously.^[^ ^62^ ^]^ Brains were removed and the optic chiasm exposed in whole mount under a fluorescence dissecting microscope. Fertilized chicken eggs were incubated for 3 days at 38.5 °C under standard conditions. Plasmidic DNA solutions were injected in the lumen of the chick neural tube as previously described.^[^ ^35^ ^]^ Chicken embryos were incubated for two more days before analysis.

### Cell Culture Assay

For luciferase assay, HEK293 cells were grown in 90% DMEM 10% fetal calf serum supplemented with 2 mm glutamine and penicillin/streptomycin (100 UmL^−1^ to 100 µg mL^−1^) (Invitrogen). HEK293 cells were transfected using LipoD293 (SignaGen) with efficiency larger than 90%. Plasmids were transfected in a ratio of 1:1:0.1 (construct of interest in pGL3 basic: Pou4f1 in pCDN3: *β*‐globine reporter) with a total DNA amount of 1 µg per 100 000 cells. The luciferase reporter plasmid used in this study was pGL3basic. Luciferase activity was measured at 24 h after transfection, using the Dual Luciferase Assay kit (Promega).

### In Situ Hybridization and Immunohistochemistry

E16.5 mouse embryos were extracted from the pregnant mother and intracardially perfused with 4% paraformaldehyde. Mouse heads were post‐fixed in the same fixative for 4 h and washed in PBS three times. The tissue was cryoprotected in 30% (w/v) sucrose in PBS. Once cryoprotected, the samples were included in OCT compound, frozen and stored at −80 °C until use. Coronal sections (20 µm) were obtained with a cryostat (SLEE medical GmbH, Mainz) and stored at −20 °C until used. Electroporated postnatal mice were transcardially perfused with PBS and 4% paraformaldehyde. Brains were dissected out and post‐fixed in the same fixative overnight and washed in PBS. Coronal sections (80 µm) were obtained with a vibratome (Leica). The spinal cord from electroporated chicken embryos were immersion fixed in 4% paraformaldehyde for 2 h, washed in PBS, and sectioned with a vibratome (Leica). In situ hybridization was performed according to reported methods.^[^ ^63^ ^]^ A riboprobe to detect mouse Nrp2 mRNA was kindly provided by Dr. Lynda Erskine.

For immunohistochemistry, antigen retrieval with Citrate Buffer pH 6 during 10 min at RT. After that, they were incubated during 20 min with Citrate Buffer at 90 °C. After two washes with PBS, sections were treated with MetOH at 10% during 20 min Sections were blocked for 1 h in PBS containing 5% fetal bovine serum, 0.25% Triton‐X‐100, and gelatine at 0.02%. Sections were incubated overnight at 4 °C in blocking solution with the corresponding primary antibodies. The following primary antibodies were used: chicken anti‐GFP (Aves Labs); mouse anti‐Pou4f1 (Millipore); rabbit anti‐Sncg (GeneTex); mouse anti‐Sncg (Abnova); rabbit anti‐Zic2 (Herrera's lab.^[^ ^64^, ^65^, ^66^, ^67^ ^]^ For immunofluorescence detection, Alexa 488, Alexa 546, and Alexa 647 (Invitrogen, Molecular Probes) secondary antibodies were used. Nuclei were counterstained with DAPI (2 µg mL^−1^).

### Microscopy Setup

Images were captured with an Olympus FV1000 confocal IX81 microscope/FV10‐ASW software. Chiasm images were acquired using a Leica MZ16F stereoscope. Electroporated cortex images were acquired with a slide‐scanner Zeiss Axioscan.

### Quantification and Statistical Analysis

Data were presented in a form of mean ± SEM. To quantify retinal projections at the optic chiasm level, squared regions of interest (ROI) were superimposed on the width of the optic nerve close to the electroporated retina, the opposite optic nerve, the contralateral optic tract, and the ipsilateral optic tract in regions proximal to the chiasm. Fluorescence intensity within each ROI was measured using Fiji software and normalized with respect to the background. The percentage of fluorescence intensity in each ROI relative to the optic nerve ROI on the electroporated side was then represented graphically. Quantification in spinal and brain sections followed the same strategy: ROIs were drawn in the ipsilateral and contralateral regions of projecting axons. SPSS, GraphPad Prism 8.0, or R (version 4.0.4) was adopted for statistical analyses. Two groups were compared by unpaired, two‐tailed, Student's *t*‐tests and three groups were compared by one‐way ANOVA with Bonferroni tests. Statistical significance was calculated according to the log‐rank test *p* ≤ 0.05 stood for statistical significance (*p < 0.05; **p < 0.01; ***p < 0.001).

## Conflict of Interest

The authors declare no conflict of interest.

## Authors Contribution

M.F.N. performed the RNA‐seq and ATAC‐seq wet experiments. M.T.L.‐C., J.F.‐A., and R.M.‐V. performed the computational analysis of RNA‐seq and ATAC‐seq data. A.E. performed electroporation experiments in chick and cortex. V.M.B. performed ISH for Nrp2 in Zic2 electroporated embryos. E.H. wrote the original draft and designed, conceived, and supervised the study. A.B. co‐wrote the manuscript, helped with the experimental design, and critically revised the manuscript for important intellectual content.

## Supporting information

Supporting Information

Supporting Information

## Data Availability

Genomic data sets can be accessed at the GEO public repository using the accession number GSE184275. The previously published ChIP–seq data re‐analyzed in this study are available under accession code GSE133492
