# Supplementary material for: Multiomic Analysis of Neurons with Divergent Projection Patterns Identifies Novel Regulators of Axon Pathfinding
Source: Adv Sci (Weinh). 2022 Aug 21;9(29):2200615. doi: 10.1002/advs.202200615 (PMC9561852; doi:10.1002/advs.202200615)
Supplement: Supplementary file 1 — Supporting Information [file ADVS-9-2200615-s002.pdf]

Figure Legends to Supplementary Figures

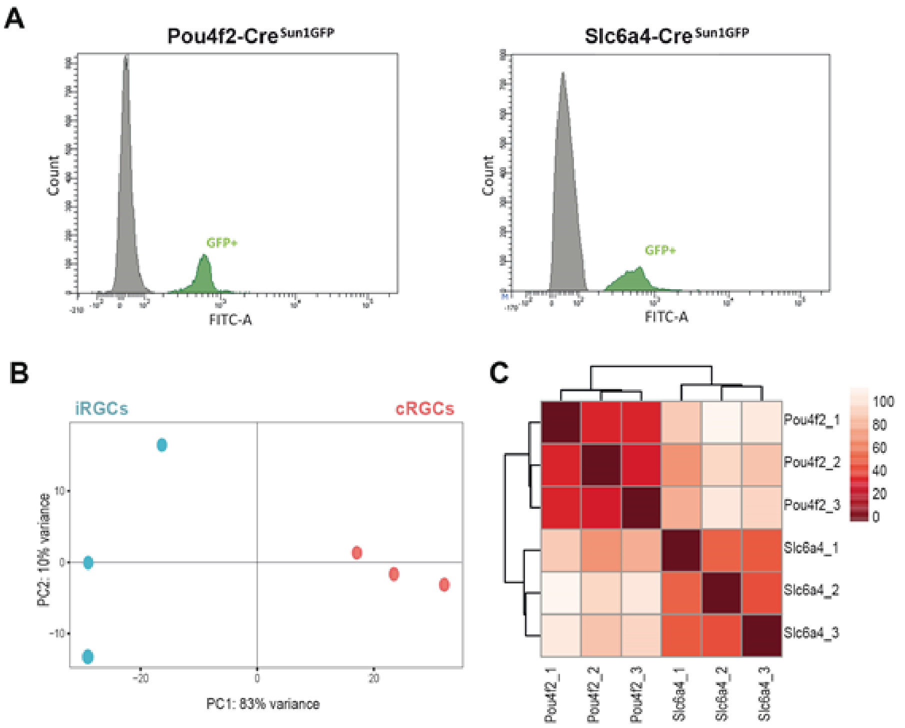

Figure S1

Figure S1. A. Flow cytometry signal for the specific isolation of fluorescence Sun1-GFP^{+} cells from retinas of Slc6a4^{SunGFP} and Pou4f2^{SunGFP} embryos.

B. Principal component analysis of RNA-seq samples. Slc6a4^{SunGFP} n=3; Pou4f2^{SunGFP} n=3 independent samples.

C. Pearson correlation matrix between normalized RNA-seq samples clustered by Euclidian dendrogram. Slc6a4^{SunGFP} n=3; Pou4f2^{SunGFP} n=3 independent samples.

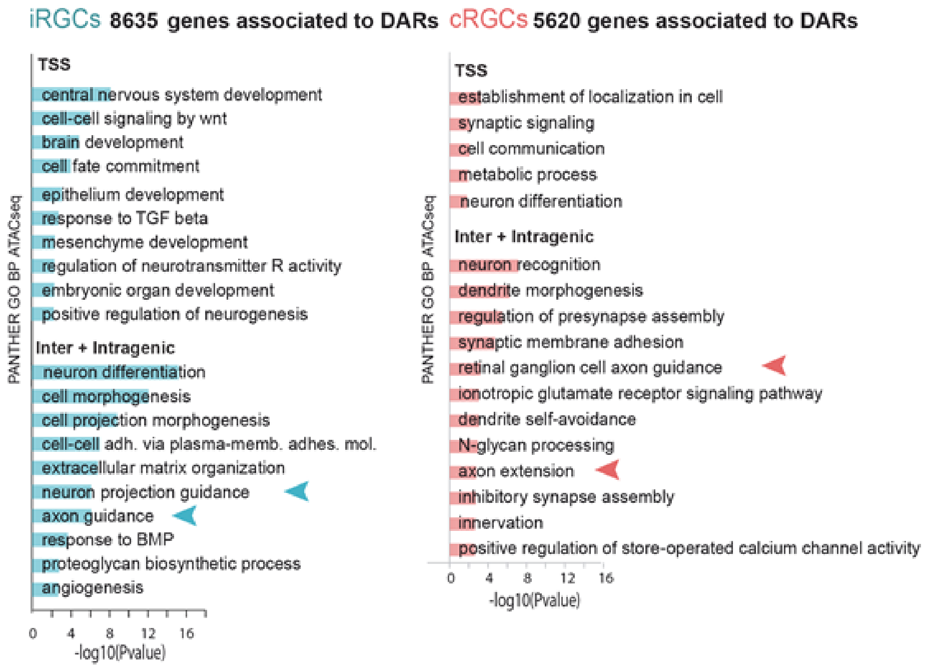
iRGCs 8635 genes associated to DARs
cRGCs 5620 genes associated to DARs
TSS
central nervous system development
cell-cell signaling by wnt
brain development
cell fate commitment
epithelium development
response to TGF beta
mesenchyme development
regulation of neurotransmitter R activity
embryonic organ development
positive regulation of neurogenesis
Inter + Intragenic
neuron differentiation
cell morphogenesis
cell projection morphogenesis
cell-cell adh. via plasma-memb. adhes. mol.
extracellular matrix organization
neuron projection guidance
axon guidance
response to BMP
proteoglycan biosynthetic process
angiogenesis
PANTHER GO BP ATACseq
0
4
8
12
16
-log10(Pvalue)
TSS
establishment of localization in cell
synaptic signaling
cell communication
metabolic process
neuron differentiation
Inter + Intragenic
neuron recognition
dendrite morphogenesis
regulation of presynapse assembly
synaptic membrane adhesion
retinal ganglion cell axon guidance
ionotropic glutamate receptor signaling pathway
dendrite self-avoidance
N-glycan processing
axon extension
inhibitory synapse assembly
innervation
positive regulation of store-operated calcium channel activity
PANTHER GO BP ATACseq
0
4
8
12
16
-log10(Pvalue)

Figure S2

Figure S2. Panther GO Biological Process enrichment analysis of DARs located in the TSS or in enhancers that include both distal and proximal cis regulatory elements (CRE) and intragenic regions in cRGCs and iRGCs. P.adj < 0.05 and $|\log2FC| \geq 1$.

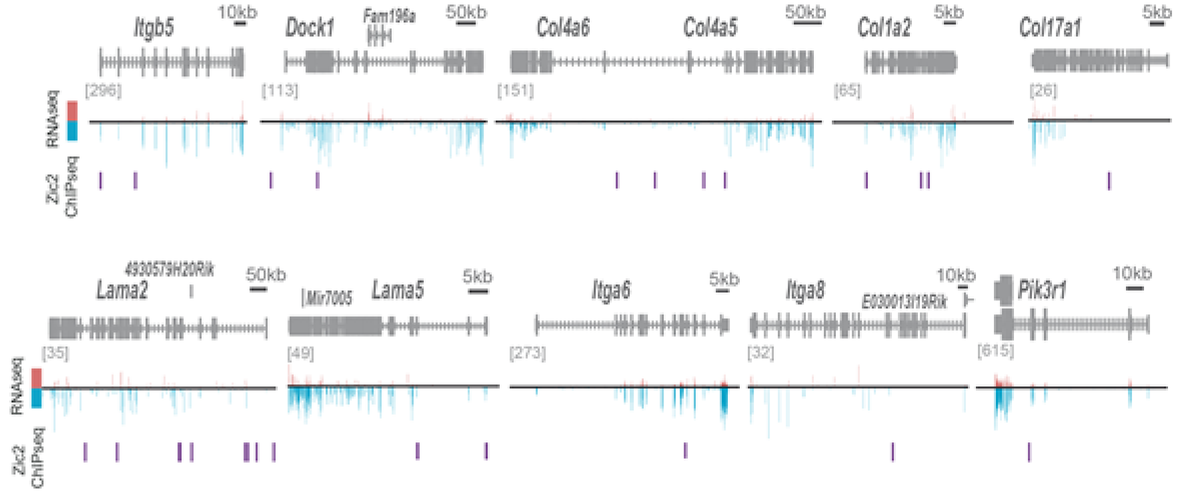

Figure S4

Figure S3. Genomic snapshots of RNA-seq and Zic2 ChIP-seq in retina at loci that encode for integrins differentially expressed in iRGCs and cRGCs.
